# Awareness and Stimulus-Driven Spatial Attention as Independent Processes

**DOI:** 10.3389/fnhum.2020.00352

**Published:** 2020-09-02

**Authors:** Diane Baier, Florian Goller, Ulrich Ansorge

**Affiliations:** ^1^Department of Cognition, Emotion, and Methods in Psychology, Faculty of Psychology, University of Vienna, Vienna, Austria; ^2^Department of Consumer Service, University of Applied Sciences Wiener Neustadt, Wiener Neustadt, Austria; ^3^Cognitive Research Hub, University of Vienna, Vienna, Austria

**Keywords:** awareness, stimulus-driven attention, spatial attention, metacontrast masking, singleton

## Abstract

To investigate the relation between attention and awareness, we manipulated visibility/awareness and stimulus-driven attention capture among metacontrast-masked visual stimuli. By varying the time interval between target and mask, we manipulated target visibility measured as target discrimination accuracies (ACCs; Experiments 1 and 2) and as subjective awareness ratings (Experiment 3). To modulate stimulus-driven attention capture, we presented the masked target either as a color-singleton (the target stands out by its unique color among homogeneously colored non-singletons), as a non-singleton together with a distractor singleton elsewhere (an irrelevant distractor has a unique color, whereas the target is colored like the other stimuli) or without a singleton (no stimulus stands out; only in Experiment 1). As color singletons capture attention in a stimulus-driven way, we expected target visibility/discrimination performance to be best for target singletons and worst with distractor singletons. In Experiments 1 and 2, we confirmed that the masking interval and the singleton manipulation influenced ACCs in an independent way and that attention capture by the singletons, with facilitated performance in target-singleton compared to distractor-singleton conditions, was found regardless of the interval-induced (in-)visibility of the targets. In Experiment 1, we also confirmed that attention capture was the same among participants with worse and better visibility/discrimination performance. In Experiment 2, we confirmed attention capture by color singletons with better discrimination performance for probes presented at singleton position, compared to other positions. Finally, in Experiment 3, we found that attention capture by target singletons also increased target awareness and that this capture effect on subjective awareness was independent of the effect of the masking interval, too. Together, results provide new evidence that stimulus-driven attention and awareness operate independently from one another and that stimulus-driven attention capture can precede awareness.

## Introduction

Up until today, the relationship between attention and awareness is debated. This is also true of vision. On the one hand, attention could operate following awareness and even depend on it (cf. Shiu and Pashler, 1995; Ward et al., 2016). On the other hand, attention could be critical for visual consciousness or awareness (Titchener, 1908; Scharlau and Neumann, 2003; cf. Chica et al., 2010, 2011).

The former position is in line with the assumption of a rich visual representation that is not limited to only few details, but instead initially and automatically covers more information than maybe a limited ability to report visual information would suggest (Lamme, 2003; see also Bronfman et al., 2014, 2019). Corresponding theories do not deny attentional selectivity, but rather put the processing bottleneck accounting for selectivity at a later stage of processing, for example, during transfer of information to working memory or during (selection of information for) report (Sperling, 1960; Wolfe, 1999; Lamme, 2003, 2006; Sligte et al., 2008; Vandenbroucke et al., 2014; see also Usher et al., 2018).

According to the alternative position, the processing bottleneck is located at a very early stage, before information even reaches awareness. One of the early filter mechanisms is stimulus-driven attention (Theeuwes, 1992; Donk and van Zoest, 2008; Mulckhuyse and Theeuwes, 2010; cf. Chica et al., 2012). Stimuli can draw our attention in two different ways (Egeth and Yantis, 1997; Wolfe et al., 2003; Connor et al., 2004; Weichselbaum et al., 2018): in an intentional, goal-directed way (top–down/endogenous) or in an automatic, stimulus-driven way (bottom–up/exogenous). In top–down capture, only a stimulus matching the goals and/or search intentions of the observer will capture attention; for example, when looking for a tomato in the supermarket, only red and round objects will be selected for further processing (e.g., Duncan and Humphreys, 1989; Folk et al., 1992; Büsel et al., 2018). Irrelevant stimuli, however, are suppressed and ignored (e.g., inattentional blindness; Mack and Rock, 1998; Eitam et al., 2013; Horstmann and Ansorge, 2016). In contrast, in stimulus-driven capture, stimuli capture attention by standing out among other stimuli or against the background (e.g., Nothdurft, 1993), for example, due to their overall salience (i.e., their local differences in color, intensity, and orientation; Itti and Koch, 2001). For example, a color singleton, with a unique color presented among color-homogeneous non-singletons, would capture attention even if task-irrelevant (Theeuwes, 1992; Weichselbaum et al., 2018). The selected stimuli are attended to, and the corresponding information is maintained in visual short-term memory (STM), whereas unattended stimuli are not processed further, and the information is lost (Baddeley, 1986).

Hence, in the early-selection approach, attention is seen as a gateway to awareness (e.g., Wundt, 1896; Posner, 1980; Mack and Rock, 1998; Ambinder and Simons, 2005; Neumann and Scharlau, 2007; Asplund et al., 2010). Classic theories of automatic processing confirm that stimulus-driven attention works independently of awareness (e.g., McCormick, 1997; Mulckhuyse et al., 2007; Schöberl et al., 2015).

Yet, although attention is sometimes assumed to be a prerequisite for awareness (e.g., Mack and Rock, 1998; Ambinder and Simons, 2005), this does not mean that attention mandatorily entails awareness (Lamme, 2003). For example, subliminally presented visual stimuli, that is, stimuli below the threshold of visual awareness, can capture attention in a stimulus-driven or goal-directed way, without eliciting participants’ awareness of these stimuli itself (e.g., Kentridge et al., 1999, 2004; Scharlau and Ansorge, 2003; Ansorge et al., 2009, 2010; Schöberl et al., 2015). In addition, elective attention can speed up fading of stimulus-related sensory experience (e.g., Bachmann and Murd, 2010; Murd and Bachmann, 2011), which speaks against attention as the sole or even the main cause of awareness.

The big challenge—and possibly the reason for the still unresolved debate about the relation between attention and awareness—is to convincingly measure awareness. Some kind of report or “direct measure” of awareness is required to obtain results (see Reingold and Merikle, 1988). However, it is often hard to determine if deficient report goes back to a lack of awareness, or if another process crucial for report was impaired (e.g., transfer to working memory or access consciousness) and thereby diminished performance on a direct measure (Eriksen, 1960; Block, 2011).

To tackle this problem, different experimental approaches have been taken. However, each of these approaches entails complications, some of which we describe in the next section. Here, to solve them, we chose a new approach: a combination of metacontrast-masking and stimulus-driven attention by color singletons. In visual masking, the visibility of one stimulus—the target—is reduced by a subsequently presented stimulus—the mask (Breitmeyer et al., 1984; Bachmann, 1994; Enns and Di Lollo, 2000). In metacontrast masking, both temporal vicinity and spatial vicinity of mask and target jointly diminish target visibility (Stigler, 1910; Breitmeyer and Öğmen, 2006). The inner contours of the mask have to (closely) surround the outer contours of the target (corresponding to spatial vicinity), and the stimulus-onset asynchrony (SOA) between target and temporally trailing mask must be small (corresponding to temporal vicinity). Typically, metacontrast masking is strongest if the SOA exceeds zero by some tens of milliseconds (e.g., about 30–60 ms in Alpern, 1953), provided that the ratio of the energy of the mask (luminance times duration) and that of the target are not too big. The combination of these factors leads to a diminished visibility and awareness of the target (cf. Kentridge et al., 2008).

### Evidence for Attention-Independent Awareness and Its Relation to Iconic Memory

Hitherto, results supporting the assumption that a rich awareness precedes the operation of attention stem mostly from iconic memory investigations (Sperling, 1960; Hanning et al., 2015; Mack et al., 2016). In iconic memory research, several rows of letters are presented for a short time. Afterward, a post-cue points out the relevant stimuli and draws attention to them. As the performance for selected items in this partial report scenario is better compared to the proportion of correctly recalled items during full report of all stimuli, authors concluded that initial awareness of stimuli represented in iconic memory is rich, and attention occurs at a later stage serving as a gateway for stimuli to access working memory in order to be reported (Sperling, 1960). Since Sperling’s (1960) research, many studies confirmed influences of attention on iconic memory representations (Mack et al., 2015, 2016, 2019).

However, Mack and colleagues’ conclusion that awareness requires attention has been subject to critique: Aru and Bachmann (2017a; see also Bachmann and Aru, 2015, 2016) found evidence for the existence of autonomous awareness; their results show that attention is a different process, even if it has an effect on awareness. In addition, to prove attention-independent effects, the choice of letters as items for iconic-memory report is not ideal, as processing of letters is—depending on their context—a conjunction task, and thus, a special type of visual awareness (i.e., for conjunctions of features within one object) is required (cf. Treisman, 1977; Treisman and Gelade, 1980).

### Current Approach

To avoid an artificial boost of post-perceptual attentional influences, in Experiments 1 and 2, we asked our participants to report only a single feature—the position of a masked missing sector (i.e., a gap) of a disk. In addition, we used only four possible relevant stimuli to stay inside memory capacity (Sperling, 1960; Luck and Vogel, 1997). This is important to rule out that the early, awareness-independent influence of spatially selective attention that we expected could have been due to the fact that memory capacity was already exceeded. In addition, as we used near-threshold targets combined with post-target cues (indicator lines, which appeared only after the target had disappeared), we had to keep the task simple to prevent a floor effect and not being able to measure any attention-dependent influences at all.

In our Experiments 1 and 2, we manipulated target visibility by varying the exact interval between target and mask. This was done in order to present the target closer to or farther from its visibility threshold (cf. Alpern, 1953). Each stimulus in the display had a gap at one of two possible positions, and we asked our participants to discriminate the location of the gap of the one post-target cued target stimulus out of four possible stimuli. For instance, participants pressed a key at the top for a target with a gap at the top, and they pressed a key on the right for a target with a gap on the right. In Experiments 1 and 2, we tested if stimulus-driven attention capture toward the target facilitated visibility/discrimination performance. We expected that if the influence of stimulus-driven attention capture is independent of the participants’ awareness of the targets and even can precede the awareness of the targets, then the influences of our attention manipulation and that of masking on stimulus visibility should be fairly additive or independent (cf. Agaoglu et al., 2016).

Importantly, in Experiments 1 and 2, we thus used participants’ ability to objectively discriminate target shapes as a measure of target visibility and as a proxy of target awareness. Although an objective visibility measure typically yields more conservative estimates of residual awareness than the participant’s subjective report of their experienced awareness, this procedure allows for two related objections to the assumption that visibility corresponds to awareness. First, one objection is that even accurate performance under masked conditions—our visibility measure—might not reflect visibility/awareness but instead reflected either awareness-independent motor priming of the correct responses (Neumann and Klotz, 1994; cf. Kunde et al., 2003) or “correct guessing” in the absence of awareness (Weiskrantz et al., 1974; cf. Reingold and Merikle, 1988). A second, related objection is that incorrect performance might not be due to the absence of awareness but could be due to errors under aware conditions, such as erroneous button presses because the proper stimulus-response mapping has been forgotten etc. The latter objection is particularly relevant where the average residual objective discrimination performance is on average better than chance level and the “zero-discrimination” criterion of non-conscious processing is violated (cf. Eriksen, 1960; Holender, 1986; Schmidt, 2015).

To tackle these problems, we took a variety of measures. First, to prevent awareness-independent contributions of motor priming to our awareness measure, we had to avoid that, per each trial, only a single masked target could have primed a single correct response in an awareness-independent fashion (Neumann and Klotz, 1994; cf. Kunde et al., 2003). To that end, we presented four potential target stimuli per trial before a post-target cue indicated which target to report, and we always presented two of the four potential targets with one to-be discriminated, response-relevant gap location, and two stimuli of the four potential targets with the other to-be-discriminated, alternative response-relevant gap location. In this way, each target display was balanced regarding the response-relevant features, and awareness-independent response priming by only a single gap location was prevented.

Second, to address the problem that any accuracy differences could have reflected the same degree of (un-)awareness—correct guesses in the absence of awareness or different ratios of incorrect responses in the presence of awareness—we also asked our participants for their subjective awareness of the targets. This was done in Experiment 3. If our visibility measures in Experiments 1 and 2 reflected awareness, we expected to replicate major findings achieved with an objective measure also with a subjective measure of awareness. Finally, to see if differences in objective performance could have reflected accuracy differences only under aware conditions, in Experiment 1, we also tested if the influence of stimulus-driven attention on performance was present under zero-discrimination or chance-performance conditions. To that end, we regressed the individual attention-capture effects on the individual discrimination ability scores and tested if the attention-capture effect [in reaction times (RTs)] was present where objective discrimination ability was not better than chance (cf. Greenwald et al., 1996).

To manipulate stimulus salience for our test of awareness-independent stimulus-driven attention capture by a single stimulus in each display (i.e., the target or a distractor), we used the gap-independent color-singleton configuration of the target or a distractor. Where the target was a singleton, attention would have been captured by and toward the target (singleton-target condition), and better performance (higher accuracy and higher awareness) was expected. This was expected in comparison to the condition where a distractor was the singleton and would have captured attention away from the target (singleton-distractor condition), thereby compromising target processing, here its visibility (Theeuwes, 1992; cf. Becker et al., 2009).

Critically, with our color-singleton manipulation, we were careful not to repeat some complications of prior research. Most importantly, in contrast to Tata (2002), we did not use a shape-salience manipulation and a corresponding search-asymmetry effect (cf. Treisman and Souther, 1985) for our manipulation of stimulus-driven attention capture. Instead, we used a color-salience manipulation to prevent complications surrounding pop-out manipulations by shape-based search asymmetries. Search-asymmetry effects have sometimes been explained through interactions between (pooled) activities of neurons in (primary) visual cortex (cf. Li, 1999), much as some forms of visual masking themselves (e.g., Bridgeman, 2007). Because using shape-based search asymmetry as a salience manipulation therefore carries the risk of creating an interaction between salience and awareness (here: more or less masking) that is not of an attentional origin, we used a color-salience manipulation. Such color-salience manipulations are known to create more robust stimulus-driven attention capture effects than (some of the) shape-salience manipulations anyway (Theeuwes, 1992).

## Experiment 1

To examine the interaction between awareness and stimulus-driven attention, we varied the visibility of stimuli by metacontrast masking and arranged them in different singleton configurations in Experiment 1. We used color singletons (one red stimulus among green stimuli, or one green stimulus among red stimuli) to trigger stimulus-driven attention shifts (cf. Theeuwes, 1992; Weichselbaum et al., 2018). There were three different configurations: the singleton was the target (attention at the target position; target singleton), the singleton was one of the distractors (attention at a distractor position; distractor singleton), and no singleton present (half of the stimuli in one color, other half in the other color, with color changes between all adjacent positions). In the latter setup, there is, thus, no singleton present.

For the factor singleton configuration, we expected different levels of awareness, here measured as target visibility in a direct objective discrimination measure: higher awareness (fewer errors in the discrimination of the location of the gap of the masked target) when the singleton was the target, lower target visibility or awareness (more errors) when the singleton was one of the distractors, and neither facilitation nor inhibition of target visibility or awareness when there was no singleton present (e.g., Lamy and Egeth, 2003).

To ensure that the expected singleton-configuration effect was indeed due to stimulus-driven attention capture, we took the following measures. First, the position of the color singleton among the four potential target disks was not predictive of the target position. Across trials, the singleton position and the target position were uncorrelated (resulting in 75% distractor-singleton and in 25% target-singleton conditions). Second, for the participants, it was neither necessary to search for the color singletons to find the targets (as the targets were indicated by a post-target cue/indicator line) nor to decide which response was to be given (as the required response was indicated by the location of the missing gap inside the target). Third, even the color of the target changed unpredictably from trial to trial, meaning color search was also not possible.

The effect of stimulus-driven attention that is independent and thus also at work before awareness was tested with a criterion for awareness-independence, which did not depend that much on zero-discrimination of the respective stimuli. Typically, one would use a zero-discrimination criterion of a target and show that attention would operate even when participants are not able to discriminate the target with above-chance probability (cf. Neumann and Klotz, 1994). Such zero discrimination, however, is difficult to show where the manipulation in question increases visibility and the participants’ awareness of the stimulus itself. In our case, this concerns the manipulation of attention-capture by the to-be-discriminated stimulus (capture or no capture; see prediction for target-singleton configuration, above). Thus, we demonstrated an awareness-independent effect of stimulus-driven attention with a criterion that did not depend on zero discrimination.

We used a known modulator of target visibility in backward masking—the SOA between target and mask—to manipulate stimulus awareness and to show that singleton configuration influenced target discrimination independently of the variable SOA. We included five different SOAs, from simultaneous presentation of disks (used as target and distractors) and surrounding rings as masks (with one mask surrounding each disk) up to 289 ms to replicate the characteristic u-shaped function of visibility or awareness (i.e., judgment accuracies or stimulus visibility) in metacontrast masking (e.g., Alpern, 1953; Enns and Di Lollo, 2000). If stimulus-driven attention and visibility/awareness work independently, we would expect no statistical interaction between the different singleton configurations and the levels of the masking interval (cf. Agaoglu et al., 2016). If, however, we find differences in the strength of stimulus-driven capture by singletons depending on the level of stimulus visibility/awareness (e.g., stronger singleton capture for better discriminated stimuli, like it has been shown with goal-directed attention; Simione et al., 2019), this would speak for some kind of dependency between stimulus-driven attention on the one hand and the level of visibility/awareness on the other hand.

In addition, we also looked at the singleton-configuration effect—here, the performance difference between distractor-singleton and target-singleton configurations—at the most effective SOA (where masking was the strongest) as a function of the individual discrimination ability. We expected that a visibility- or awareness-independent effect of attention should be on average independent of the participants’ discrimination abilities. This should be reflected in a non-significant slope of a linear regression of individual singleton-configuration RT effects on individual discrimination performances and an above-chance singleton-configuration RT effect at zero discrimination ability in this regression (cf. Greenwald et al., 1996). In contrast, a singleton-configuration RT effect that depends on awareness was expected to increase with increasing discrimination ability, and it should not be present at the point of zero discrimination.

Finally, we also split our sample of participants into half on the basis of the individual discrimination performance in the most difficult neutral SOA condition and tested for the singleton-configuration effect between the resulting groups. If the singleton-configuration effect is independent of stimulus awareness, we expected to see it among the good and among the bad discrimination performers. If, however, the singleton-configuration effect depended on stimulus awareness, we expected to see a stronger or maybe selective singleton effect among the good discrimination performers.

Additionally, we also varied the intensity of the masks by using tight-fitting masks as well as loose-fitting ones. We expected tight-fitting masks to better mask the disks than loose-fitting masks (cf. Bridgeman and Leff, 1979). We manipulated the fit of the masks around the targets (and distractors) to address potential interactions among early visual cortical neurons. If our manipulation of stimulus-driven capture was responsible for the expected better visibility (or less masking) of targets under salient-target conditions than under salient-distractor conditions, we expected no interaction between the manipulation of salience and the fit of the masks. However, if our salience manipulation would have exerted its effect via some non-attentional interactions between neurons in visual cortical areas concerned with mask and/or target processing, we would have expected a significant interaction between fit of the mask and the salience manipulation.

### Materials and Methods

#### Participants

Twenty-five psychology students from the University of Vienna participated in Experiment 1 (mean_*age*_ = 22.32 years, *SD*_*a*__*ge*_ = 2.75 years) and received course credit for their participation. Their vision and color vision were normal or corrected to normal. The participants were treated in line with established ethical standards. Prior to testing, they were thoroughly instructed and signed an informed consent form. They knew that they could always abort the experiment without negative consequences, and their well-being was closely monitored throughout testing. At the end, all participants were debriefed orally and in writing.

#### Apparatus and Stimuli

**FIGURE 1:**
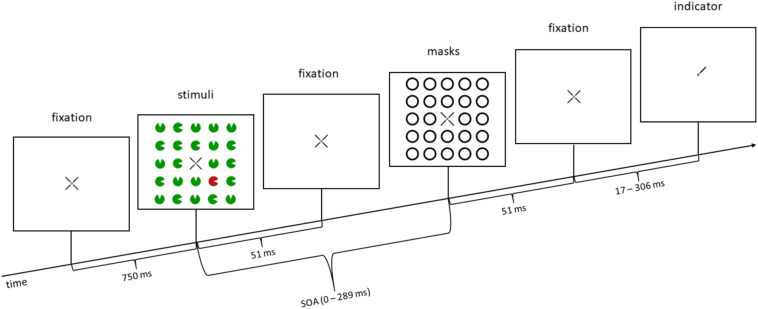
Example trial: in this trial, a color singleton is presented (a red disk in the lower right corner; second panel from the left), but it is a distractor, not the target (distractor singleton, or invalid condition for the additional task in Experiment 2), as the target is in the upper right corner (indicated by the line pointer of the fixation cross in the rightmost panel). In the illustrated trial, in Experiment 1, participants would press the upper key, as the position of the missing minor sector (gap location) of the relevant disk in the upper right corner is at the top. In Experiment 2, participants would additionally react to the line type of the pointer (rightmost panel), before reacting to the missing minor sector. In this example, the line type would be solid (as opposed to dashed; for further details, see section “Materials and Methods” of Experiment 2). SOA, stimulus-onset asynchrony. The arrow depicts the flow of time. Stimuli are not drawn to scale.

The stimuli were 24 colored disks (major circular sectors), with one missing minor sector each (gap; Figure 1). They were arranged in a square shape enclosing the fixation cross in the middle of the screen. The fixation cross consisted of a small dot (0.2° visual angle) and four indicator lines (1° × 0.1° visual angle) arranged around it (Figure 1). The four indicator lines pointed to the relevant four potential target disks in the inner corners of the square. The diameter of the circles was 1.2° visual angle. The ring-shaped masks were 0.2° visual angle thick, and the loose masks had a distance of 0.2° visual angle to the disks. The distance between the center of the four relevant disks and the center of the screen was 3.1° visual angle. The distance between disks (center-to-center, horizontally, and vertically) was 2.2° visual angle. The colors of the stimuli were green (CIE *L*^∗^*a*^∗^*b*^∗^, 38.2/−41.3/35.1) and red (38.4/52.0/24.7). Both colors had the same distance (Δ*E*_*red*_ = 70.2, Δ*E*_*green*_ = 96.8) to black (1.1/−0.7/−1.2). Note that red and green were equiluminant. Stimuli were presented against a gray background (80.9/8.6/0.2) on 24.5-inch LCD monitors (AOC G2590PX; resolution 1,920 × 1,080 pixels, vertical refresh rate 100 Hz) with an Intel HD (Graphics 630, GT2, 64/128-bit color) graphics card. Participants sat centrally in front of the monitor. The distance between their eyes and the screen was kept constant at 50 cm by a chinrest. Small lamps dimly lit the room. Up to five participants were tested at a time. To minimize visual distraction, every participant sat at a single desk, separated from the other desks by partitioning walls. Participants wore earmuffs in order to prevent auditory distraction. They responded by pressing keys on a standard keyboard with one of their index fingers. The experiment was programmed and conducted using E-Prime 2.0 (Psychology Software Tools, Pittsburgh, PA). Data were analyzed using R (R Core Team, 2019) and the following packages: apa (Gromer, 2017), ez (Lawrence, 2016), and ggplot2 (Wickham, 2009).

#### Task and Design

Experiment 1 consisted of three within-participant variables: SOA (five steps: 0/85/153/221/289 ms), mask fit (tight fit/loose fit), and singleton configuration (target singleton/distractor singleton/no singleton). The first of the five SOA steps was a simultaneous condition, where the target, distractors, and the masks were presented together for 51 ms. In the other four conditions, the target and distractors were presented for 51 ms, separated from the masks that appeared after a fixation cross, which was presented during the interstimulus interval for the respective amount of time between stimuli [e.g., for the 34 ms following the target + distractors (which both were shown for 51 ms) in the 85-ms SOA condition]. Actually, the zero-SOA condition does not just differ from the other conditions in terms of the time that passes between disks and rings. It also differs from all other conditions by the number of sequentially presented visual transients: With an SOA of zero, there are two visual transients less, as the disks (target and distractors) and rings (masks) start and end at the same time. We expected a u-shaped distribution of the accuracy rates as a function of the different SOA steps (e.g., Alpern, 1953; Enns and Di Lollo, 2000; Tata, 2002; Boyer and Ro, 2007; Bacon et al., 2013; Agaoglu et al., 2018). We presented 24 stimuli, whereof only four (in the inner corners of the virtual square) were potential targets (highlighted by lines pointing toward these positions; Figure 1). We included more than only the three non-singletons resulting from the four potential target positions to increase the salience of the singleton configuration (see, e.g., Itti et al., 1998; Lamy and Egeth, 2003; Becker and Ansorge, 2013). In the condition where a singleton was present, one of the four stimuli in the inner corners had a different color from the 23 other stimuli. In the condition where no singleton was present, red and green stimuli alternated. We expected highest accuracies in the condition with a target singleton, lowest accuracies with a distractor singleton, and accuracies in between for the condition without singletons (cf. Lamy and Egeth, 2003). For the variable mask fit, we expected better visibility resulting in higher accuracies for the loose mask, and lower visibility as well as lower accuracies for a tight mask (Bridgeman and Leff, 1979; Schmidt et al., 2006). In addition, we expected no statistical interaction between SOA and singleton configuration, which would confirm that awareness and stimulus-driven attention work independently.

#### Procedure

At first, every participant completed 60 practice trials with increasing speed and difficulty. The ensuing experiment consisted of two blocks, one block without color singletons (240 trials), and one block with color singletons (640 trials), where the singleton was the target in 25% of the trials (non-predictive), randomly intermixed with trials where the singleton was a distractor and not the target (in 75% of the trials). Across trials, singleton positions and target positions were uncorrelated. Note that this meant that the location of the singleton was not predictive of the target position. Each trial started with the presentation of the fixation cross in the middle of the screen for 750 ms, followed by the 24 colored disks for 51 ms. After an SOA, which lasted between 0 and 289 ms, the masks were presented for 51 ms. In the simultaneous condition (SOA = 0 ms), stimuli and masks were presented simultaneously for 51 ms. After the masks, a fixation cross was shown for a different amount of time to offset the different trial lengths caused by the different SOAs between stimuli and masks, in order to keep the time between stimuli onset and indicator onset constant over trials. At last, three of the four indicator lines of the fixation cross vanished and left one indicator pointing to the specific location of the preceding target. Participants now responded by keypress to the location of the missing gap of the target disk. The target appeared randomly and equally often at the four possible positions (inner corners of the virtual square of 24 stimuli). The target and each potential distractor disk missed a minor sector each. Every participant had to choose between two adjacent positions of such missing minor sectors or gaps of the target disks (counterbalanced across participants): top versus right, right versus bottom, bottom versus left, or left versus top. The response keys were the numbers 2, 4, 6, and 8 on the number pad, representing the locations top, right, bottom, and left. Participants were asked to respond correctly and quickly.

### Results

**FIGURE 2:**
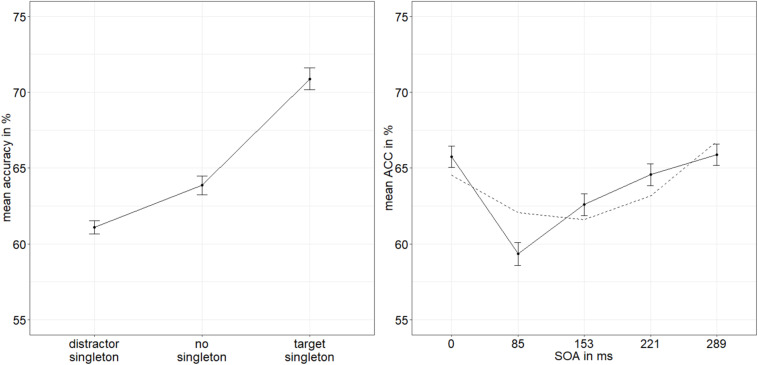
Mean accuracies (ACCs, in percent) of discriminating the position of the missing minor sector (or gap) of the target disk in Experiment 1, depending on the variables singleton configuration **(left panel)** and stimulus-onset asynchrony (SOA; **right panel**). The dashed line represents the quadratic function modeled after the ACC distribution. Error bars represent average SEs.

As an objective direct measure of visibility and as a proxy of stimulus awareness, we analyzed accuracy rates (ACCs) of the judgments about the within-disk locations of the gaps of the target disks. The ACCs were arcsine transformed. To control for potential differences of red versus green stimuli, we compared the ACCs in a *t*-test (red: 63.7%, green: 63.6%). As expected, there was no significant difference, *t*(24) = −0.15, *p* = 0.880, *d* = −0.03. We conducted an analysis of variance (ANOVA), with three within-participant variables: SOA (0/85/153/221/289 ms), mask fit (tight/loose), and singleton configuration (target singleton/distractor singleton/no singleton). Where necessary, because of a violation of the sphericity assumption, the degrees of freedom were corrected using the Greenhouse–Geisser procedure. We found significant main effects for SOA, *F*(4,96) = 10.06, *p* < 0.001, $\eta_{\text{p}}^{2}$ = 0.30, and singleton configuration, *F*(1.31,31.53) = 14.03, *p* < 0.001, $\eta_{\text{p}}^{2}$ = 0.37 (Figure 2). No further effects were found, all *p*’s > 0.132, all *s* < 0.07 (see Appendix A Table A1 for a complete listing of the ANOVA results). *Post hoc t*-tests examining the main effect of singleton configuration (α = 0.017; Bonferroni-corrected for three comparisons) showed that all differences are significant: The accuracy was higher when the target was the singleton (70.9%) than when a distractor was the singleton (61.1%), *t*(24) = 4.22, *p* < 0.001, *d* = 0.83, and higher when the target was the singleton opposed to the no-singleton condition (63.9%), *t*(24) = 3.32, *p* = 0.003, *d* = 0.65, as well as lower with a distractor singleton opposed to when no singleton was present, *t*(24) = −2.71, *p* = 0.012, *d* = −0.53. As *post hoc* examination of the main effect of SOA, we calculated a linear model with the arcsine-transformed ACCs and the factor SOA using polynomial contrasts in order to validate the shape of the distribution. The quadratic function (u-shaped) had the best fit, *t*(24) = 1.89, *p* = 0.062. The other polynomial functions had worse fits, all *t*’s (24) < | 1.58|, all *p*’s > 0.118.

As we instructed participants not only to respond accurately, but also quickly, we included (explorative) analyses of the (RTs) in Appendix B.

**FIGURE 3:**
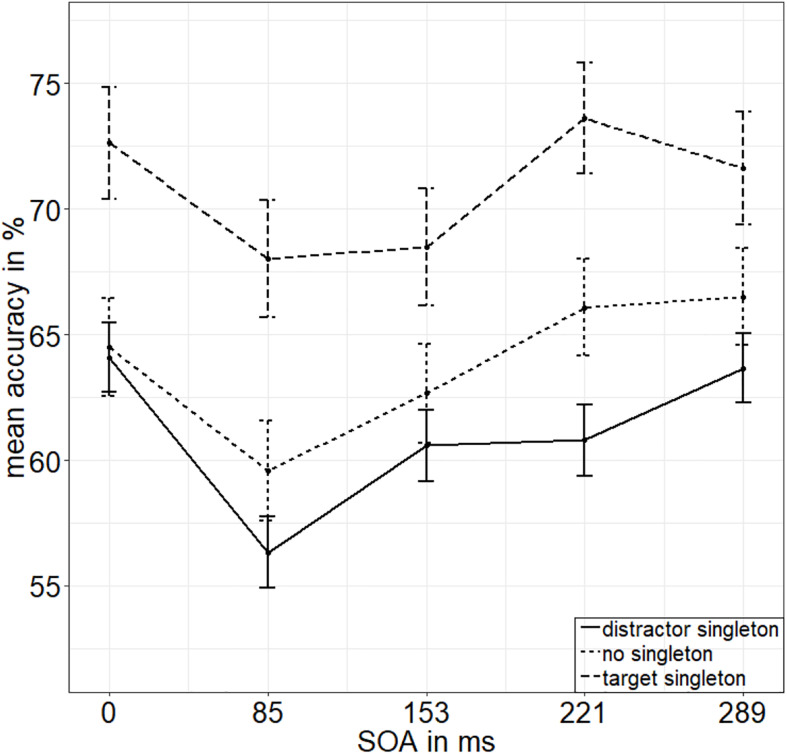
Mean accuracies (ACCs, in percent) of discriminating the position of the missing minor sector (or gap) of the target disk in Experiment 1, depending on the variables singleton configuration (line type) and stimulus-onset asynchrony (*x* axis). Error bars represent average SEs.

To calculate the value of adding the interaction between SOA and singleton configuration to the model of the main effects of those two factors, we looked at the respective Bayes factors (BFs) using JASP (JASP Team, 2019). As BFs are transitive, we divided the evidence for the main effects model against the null model (BF_10_ = 2.744 × 10^26^) by the evidence for the model with the interaction term against the null model (BF_20_ = 2.031 × 10^24^) to get the evidence for the interaction model against the main effects model (BF_12_; see van den Bergh et al., 2020). With a value of 135.106, the BF (BF_12_) shows very strong evidence for the main effects model and no evidence for the interaction effect model (BF_21_ = 0.007; Raftery, 1995). See Figure 2 for the main effects and Figure 3 for the lack of interaction between SOA and singleton configuration.

Our reasoning is that discrimination or visibility in masked conditions reflected awareness of the stimuli and that a lack of interaction between SOA and singleton configuration meant that there was the same attention-capture effect regardless of awareness. Otherwise, we would have expected the singleton-configuration effects to grow alongside with the participants’ objective discrimination performance and hence awareness (which later depended on SOA). However, as explained in the section “Introduction,” the singleton-configuration effect—better performance for target-singleton than distractor-singleton configurations—could be due to the residual awareness of the targets that was observed even in the strongest masking conditions and that could have created a singleton-configuration effect based on visible targets that for some reason did not grow any further with an increasing target visibility or awareness (i.e., more instances of the seen target). To note, this is not the most obvious prediction if residual visibility or awareness accounted for the singleton-configuration effect, as such an awareness-dependent singleton-configuration effect should have increased further with more instances of target visibility or awareness in other SOA conditions. However, we addressed the concern by incorporating two further analyses that specifically looked for evidence of stimulus-driven attention capture with less to zero-target discrimination.

**FIGURE 4:**
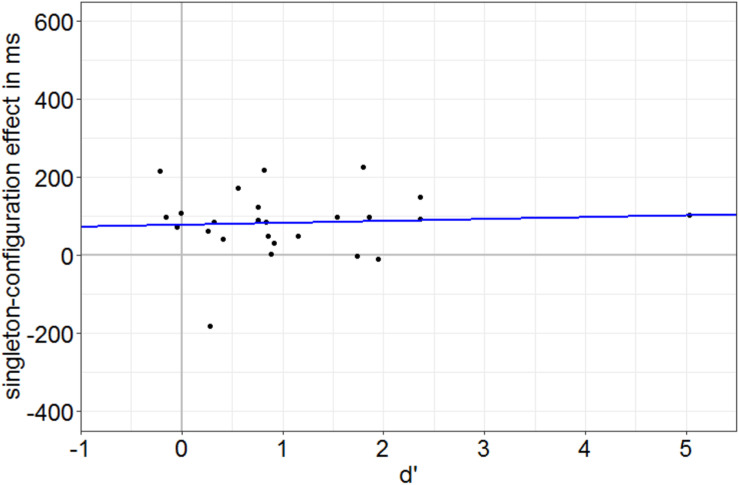
Individual singleton-configuration effect of the reaction times (RTs) in ms (RTs with distractor singleton minus RTs with target singletons) plotted against the individual discrimination value *d*’ (calculated for the neutral SOA = 85-ms condition). Each dot corresponds to one participant. The blue line represents the linear regression of singleton-configuration effects on discrimination values. Note the singleton-configuration effect of 78.4 ms at zero discrimination (intercept) and the regression line nearly parallel to the *x* axis.

First, in Experiment 1, we identified participants with a below-average discrimination performance by a median split based on the SOA condition with worst discrimination performance on average (SOA = 85 ms) in the neutral singleton condition (no singleton present). We calculated *d*′ values per person per condition by subtraction of *z*-transformed probabilities of false alarms from *z*-transformed probabilities of hits. Hits were defined as the correct key presses to one of the targets (e.g., key *8* if the missing gap was on top), whereas false alarms were defined by the same key presses (e.g., key *8*) when the alternative disk was presented (e.g., a disk with a missing gap at the right side). A *t* test confirmed that the *d*’ values of the below-average group were not significantly different from zero (i.e., fulfilled the zero-discrimination criterion), *t*(11) = 0.46, *p* = 0.657, *d* = 0.13. As the singleton-configuration effect (RT with a distractor singleton minus RT with a target singleton) was still significantly different from 0 (75.9 ms), *t*(11) = 2.40, *p* = 0.035, *d* = 0.69, these data also show that the attentional influence cannot be traced back to residual target awareness. Additional evidence for a singleton-configuration effect at zero discrimination of target gaps was found in a linear regression analysis in which individual RT singleton-configuration effects (performance in singleton-distractor minus performance in singleton-target condition) were regressed on individual discrimination at the most effective SOA. Under the assumption of an awareness-independent, awareness-preceding effect of the capture of attention, we expected a significant singleton-configuration effect at zero target discrimination and no significant slope of the regression. In line with this prediction, the RT singleton-configuration effect at the intercept (78.4 ms), which reflects zero discrimination (no awareness), is significantly different from 0, *t*(23) = 3.24, *p* = 0.004 (see Figure 4). In addition, the singleton-configuration effect is not increasing with increasing discrimination performance (as a measure of awareness), non-significant slope: *t*(23) = 0.31, *p* = 0.762. This is also visible in Figure 4: regression line approximately parallel to *x* axis. Finally, no significant difference between the singleton-configuration effects of the above- (90.7 ms) and below-average (75.9 ms) discrimination groups, *t*(16.23) = 0.42, *p* = 0.682, *d* = 0.17, was found.

### Discussion

We found the characteristic u-shaped curve of ACCs plotted as a function of the SOA, with worst performance for intermediate SOAs, and best performance for the simultaneous presentation (of stimuli and masks), as well as for long SOAs, showing that our metacontrast masking procedure to operationalize different levels of visibility (or awareness) was working as expected. The visual representations of the disks were more or less replaced by the images of the masks, depending on the different SOAs, such that stimulus awareness was varied. The singleton manipulation, capturing spatial attention toward or away from the target in a stimulus-driven way, produced effects as expected: best performance for target singletons, worst performance for distractor singletons, and intermediate performance for displays without a singleton. As no evidence for a statistical interaction between SOA and singleton configuration was found (Figure 3), while the evidence for separate main effects was very strong, we conclude that stimulus-driven attention was functioning independently of the low-level perceived processes influenced by masking. At the same time, these main effects support an influence of stimulus-driven attention by color singletons that preceded stimulus awareness and was thus even in a position to support target visibility (i.e., gate awareness of the targets; cf. Scharlau and Neumann, 2003). In addition, a regression of RT singleton-configuration effects on discrimination values and an analysis of RT singleton-configuration effects as a function of whether the individual discrimination performance was above or below median discrimination performance under the most difficult neutral discrimination conditions (SOA = 85 ms) both supported the same conclusion.

The spatial distance between target (plus distractors) and (their) surrounding mask(s) had no influence on the performance. Furthermore, as would be expected on the basis of an attentional effect of the singletons, the influence of the singleton or salience manipulation did not interact with distance, although this might not mean that much, given that the effect of distance was not significant in the first place (e.g., being too small a manipulation to create an effect on target visibility).

## Experiment 2

The results from Experiment 1 showed that stimulus-driven attention and visibility or awareness could be independent. However, we still cannot be entirely sure if the singleton effects were indeed due to the capture of spatial attention by the singletons or maybe just caused by better visibility of the singleton stimuli that was created by some other means (e.g., some form of interaction between contour and color processing at cortical levels; cf. Reeves, 1986). To clarify with a second independent measure if attention was captured by the singletons, we designed Experiment 2, in which participants additionally reacted to the line type of the indicator line (cf. Figure 1). If the singletons captured attention in a stimulus-driven way, we expected better performance under target-singleton conditions than under distractor-singleton conditions, as the to-be-discriminated indicator line was the one that pointed to the target.

In other words, the indicator (which points to the target position) is spatially closest to the target position. If this pointer is used as a probe, it should benefit from its spatial vicinity to target singletons that capture attention, and it could suffer from its spatial distance from distractor singletons—that is, the singletons could lead to spatial cueing of the target indicators. If attention was indeed captured by the singletons, we would therefore expect to find validity effects (e.g., Posner et al., 1980), namely, faster responses and fewer errors in valid conditions (singleton at indicator, i.e., in the target singleton condition) and slower responses and more errors in invalid conditions (singleton away from the indicator, i.e., in the distractor singleton condition).

In addition, we aimed at replicating the independence of SOA and singleton configuration on visibility or ACCs (regarding target gap locations; the latter as a measure of target visibility/awareness). Together with the responses to the indicator lines as probes, this procedure amounts to a dual-task protocol, and thereby, some resources were drawn away from the primary visibility or awareness measure. Note, though, that there is no particular reason to expect that this artificially (1) decreases the influence of stimulus-driven attention or (2) increases the dependency of our visibility/awareness measure on stimulus-driven attention, as the secondary indicator-line discrimination task (like the primary target-gap discrimination task) again does not imply that color or color singletons become less or more task-relevant, neither for the direct measure of awareness nor for the secondary probe-discrimination task. Again, the singleton configuration was not predictive of the target position, and it was neither necessary to search for the color singletons to find the targets or the probes (as this was indicated by a line) nor to decide which response was required [as this was indicated by the missing gap of the target in the primary task and by the indicator line (solid vs. dashed; see below) for the secondary task]. Thus, the singletons were expected to again influence performance only via stimulus-driven attention capture, and as stimulus-driven effects should not depend on the availability of mental resources (Posner and Snyder, 1975; but see Lavie, 2005), we expected to be able to replicate the effect of the singleton configuration even under the more demanding dual-task conditions of the present experiment.

### Materials and Methods

#### Participants

In Experiment 2, 24 psychology students from the University of Vienna participated (mean_*age*_ = 21.64 years, *SD*_*age*_ = 2.18 years). Two participants had to be excluded because of their error rate in the responses to the probe/indicator exceeding 20% (error rates: 28 and 56%). Two further participants were excluded, as they did not follow the instructions and responded only to the indicator but failed to give judgments concerning the direct measure of target visibility or awareness (i.e., judgments about the missing minor sectors/gap location of the target disks). Treatment of participants was the same as in Experiment 1.

#### Task and Design

In contrast to Experiment 1, we included an additional task in Experiment 2, to study if attention is captured by the singletons, with a test that is independent of the visibility of the targets. To that end, we presented the indicator line that pointed toward the targets as a probe in two versions: either solid or dashed, with equal likelihood. Participants had to discriminate the line type of the indicator line (as solid or dashed) as quickly as possible, before judging where the missing minor sector of the target disk was located (the latter as in Experiment 1; Figure 1). If we find validity effects based on the position of the singleton relative to the position of the indicator, namely, faster line-type discrimination and less errors in the valid condition (if the singleton is at the position of the indicator, i.e., in target-singleton conditions) than in invalid conditions (if the singleton is at another position than the primary-task target, i.e., in distractor-singleton conditions), this would amount to evidence for attention capture by the singletons, measured independently of target visibility.

Another difference between Experiments 1 and 2 concerned the steps of the variable singleton configuration. As we were mainly interested in the validity effects on probe RTs and the performance differences evoked by singletons at target position versus at distractor position on ACCs in the primary (target-gap) discrimination, the no-singleton condition (i.e., with alternating colors) was not included in Experiment 2—that is, we realized only target-singleton and distractor-singleton conditions. In order to grant optimal comparability, we otherwise decided to keep Experiments 1 and 2 as equal as possible.

The apparatus, stimuli, and testing environment stayed the same as in Experiment 1, except for the following changes: we used 19-inch LCD-monitors (Acer B 193; resolution 1,280 × 1,024 pixels, vertical refresh rate 75 Hz) with an Nvidia GeForce (GT 220, 32-bit color) graphics card. The colors of the target and distractor stimuli were green (CIE *L*^∗^*a*^∗^*b*^∗^, 39.2/−50.3/29.7) and red (38.6/52.2/25.3). As in Experiment 1, these colors were equiluminant, had the same distance (Δ*E*_*red*_ = 70.0, Δ*E*_*green*_ = 70.6) to black (0.9/−0.1/−1.8), and were presented against a gray background (80.0/−5.3/−18.7). The distance between the participants’ eyes and the monitors was 57 cm.

### Results

As in Experiment 1, we compared ACCs to red and green targets, to ensure that the different colors had no influence on the responses. We found no significant difference, *t*(19) = −0.59, *p* = 0.565, *d* = −0.13.

#### Target Judgments

**FIGURE 5:**
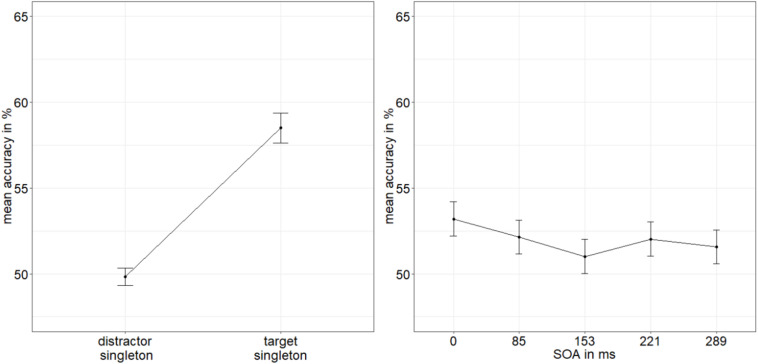
Mean accuracies (ACCs, in percent) of discriminating the location of the missing minor sectors (or gaps) of the target disks in Experiment 2, depending on the variables singleton configuration **(left panel)** and stimulus-onset asynchrony (SOA; **right panel**). Error bars represent average SEs.

We conducted an ANOVA of the arcsine-transformed ACCs, with the variables SOA (0/85/153/221/289 ms), mask fit (tight fit/loose fit), and singleton configuration (target singleton/distractor singleton). It yielded a significant main effect of singleton configuration, *F*(1,19) = 7.27, *p* = 0.021, $\eta_{\text{p}}^{2}$ = 0.11 (Figure 5), with a significantly higher accuracy rate when the target was the singleton (58.5%) than when a distractor was the singleton (49.8%). No further effects were significant, all *p*’s > 0.141, all *s* < 0.09 (see Appendix A Table A1 for all results).

As target judgments were only given after the responses to the indicator line type, RTs to the missing minor sectors were therefore not analyzed in Experiment 2.

Following the procedure from Experiment 1, we calculated the BF of the evidence for the interaction effect model versus the main effects model with the factors SOA and singleton configuration (van den Bergh et al., 2020). We divided BF_10_ = 1.177 × 10^9^ (evidence for main effects model against null effect model) by BF_20_ = 2.445 × 10^7^ (evidence for interaction effect model against null effect model). We again found strong evidence for the main effects model (BF_12_ = 48.139) and no evidence for the interaction effect model (BF_21_ = 0.021; Raftery, 1995).

#### Probe Responses to the Indicator Line Type

We additionally analyzed mean correct RTs as well as ACCs of the indicator line-type discrimination responses. For the RT analysis, RTs slower or faster than 2 SDs from the median per person per condition as well as wrong responses were excluded (13.30% in total).

**FIGURE 6:**
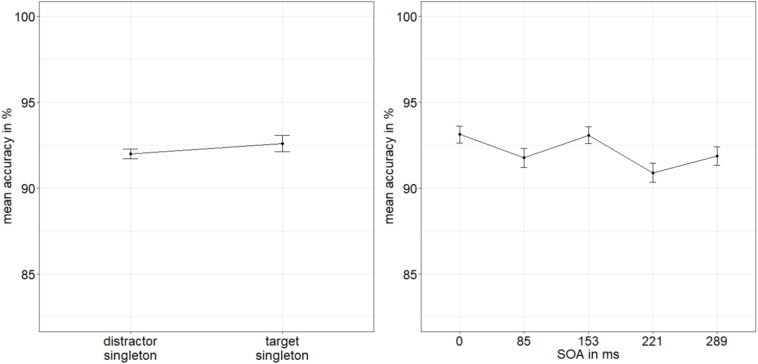
Mean accuracies (ACCs, in percent) of discriminating the line type of the indicator in Experiment 2, depending on the variables singleton configuration **(left panel)** and stimulus-onset asynchrony (SOA; **right panel**). Error bars represent average SEs.

The ANOVA of the arcsine-transformed ACCs, with the variables SOA, mask fit, and singleton configuration or validity (valid: singleton at indicator line; invalid: singleton at distractor location, yielded a significant main effect of singleton configuration or validity, *F*(1,19) = 16.24, *p* < 0.001, $\eta_{\text{p}}^{2}$ = 0.46, with significantly better performance for indicator lines in valid conditions/at singleton position (92.6%) than in invalid conditions/at a distractor position, away from the target (92.0%). No further effects were significant, all *p*’s > 0.199, all *s* < 0.09 (Figure 6; for a complete listing of all results, see Appendix A Table A2).

**FIGURE 7:**
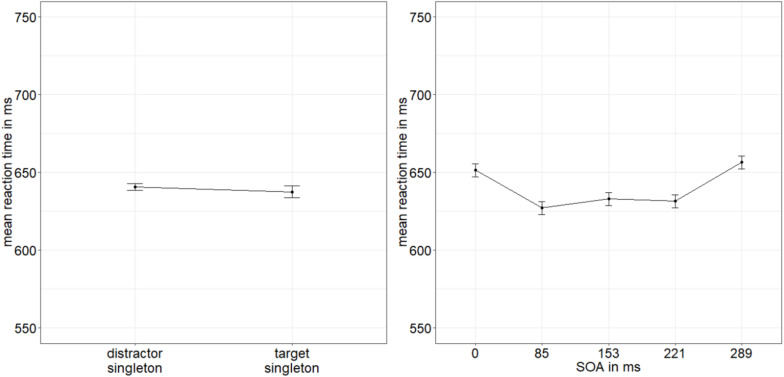
Mean correct reaction times (RTs, in ms) of the line type discrimination of the indicator in Experiment 2, depending on the variables singleton configuration **(left panel)** and stimulus-onset asynchrony (SOA; **right panel**). Error bars represent average SEs.

The same ANOVA was conducted with the mean correct RTs to the indicator lines, and it yielded a significant main effect of SOA, *F*(4,76) = 5.08, *p* = 0.001, $\eta_{\text{p}}^{2}$ = 0.21. No further effects were significant, all *p*’s > 0.073, all *s* < 0.11 (Figure 7; for a complete listing of the results, see Appendix A Table A2). *Post hoc t*-tests (α = 0.013; Bonferroni-corrected for four comparisons) examining the main effect of SOA revealed significantly slower responses with an SOA of 289 ms (mean RT: 657 ms) compared with 221 ms (mean RT: 632 ms), *t*(19) = −3.43, *p* = 0.003, *d* = −0.75, which might have reflected a hypothesis-irrelevant speed-accuracy trade-off (cf. Heitz, 2014) between these two different interval conditions. Between 51 ms (mean RT: 652 ms) and 85 ms (mean RT: 627 ms), between 85 and 135 ms (mean RT: 633 ms), and between 135 and 221 ms, there were no significant differences, all *t*s(19) < | 2.53|, all *p*’s > 0.020, all *d*’s < |0.55|.

### Discussion

Regarding the influence of the singleton configuration, the results of Experiment 2 adhere to the main findings of Experiment 1. The singleton manipulation influenced the judgments about the targets, namely, fewer erroneous judgments for target singletons and more wrong judgments for distractor singletons. Besides, the u-shaped distribution of ACCs is at least visible in the mean ACCs, although the variable SOA was not significant. A likely reason for the lacking u-shaped metacontrast function was that performance in the target judgments overall was worse (54.15%)—close to chance in many conditions of Experiment 2—than in Experiment 1 (63.65%). This flattening of the u-shaped curve around the intermediate SOAs, where the expression of the curve was limited by chance-performance levels, was most likely due to some of the resources being vied away from the primary awareness measure to the response to the indicator lines that created dual-task interference and increased the interval between the target and the judgment about the target (cf. Jackson-Nielsen et al., 2017). Again, the variation of the distance between masks and targets had no influence.

Concerning our major question, if attention was captured by the masked singletons, the additional reaction to the indicator line type produced significant validity effects in the ACCs, depending on the location of the singleton. Indicator-line discrimination was better in target singleton (valid) conditions than in distractor singleton (invalid) conditions. This shows that the singleton did indeed capture attention to its position. This finding supported our conclusion that better visibility and thus higher awareness of target singletons in the direct measure was due to stimulus-driven spatial attention.

## Experiment 3

In Experiments 1 and 2, we used an objective visibility measure as a proxy for an awareness measure. In such an objective task, however, it is theoretically possible that target discrimination performance not only reflected awareness of the targets. Errors could have also reflected the confusion of the response buttons, etc.—errors that can result despite target awareness. Typically, objective tasks are very sensitive for the residual awareness of the masked targets, even to the degree that, if the objective task is simple enough, the task could be non-exclusive for residual awareness and tap into non-conscious processing to some extent itself (Reingold and Merikle, 1988). This high sensitivity of the objective visibility tasks means that these tasks typically yield more conservative estimates of awareness-independent processing than subjective awareness tasks that only ask for the participants’ subjective awareness of the masked targets (cf. Ramsøy and Overgaard, 2004). In addition, much as it is the case for the errors in the objective discrimination tasks, errors that have little to do with target awareness are possible in a subjective task, too. In fact, the subjective report is based on a participant’s internal decision criterion about her/his awareness alone, so that there is no external yardstick by which to measure if the subjective report is valid or not. In other words, the subjective measures have to be taken at face value despite the possibility that the participants were only falsely reporting an impression of not seeing something, for example, claiming unawareness simply because of a conservative decision criterion. Nonetheless, to confirm that the basic findings of Experiments 1 and 2 hold true—a stimulus-driven attention capture effect that is additive to that of the SOA is exerted on awareness as we have assumed—we replicated our design with a subjective dependent variable: perceived stimulus awareness.

### Materials and Methods

#### Participants

Twenty-four psychology students from the University of Vienna took part in Experiment 3 (mean_*age*_ = 21.54 years, *SD*_*age*_ = 2.84 years). For information about the treatment of participants, testing environment, apparatus, and stimuli, see section “Materials and Methods” of Experiment 2.

#### Task and Design

The design was exactly the same as in Experiment 1. The task, however, was no longer to discriminate the target, but to rate the impression of awareness to the target on a scale from 1 (“not visible”) to 4 (“completely visible”).

### Results

**FIGURE 8:**
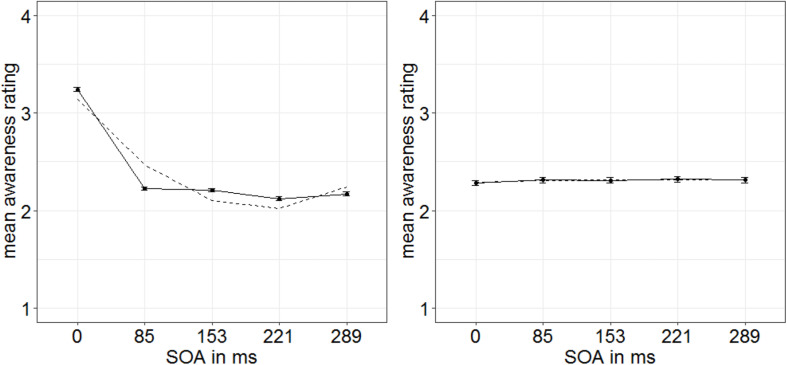
Mean awareness ratings of Experiment 3 depending on the different stimulus-onset asynchronies (SOAs) for 15 participants showing a u-shaped function (**left panel**; dashed line represents quadratic function) and nine participants showing a non–u-shaped function **(right panel)**. Error bars represent average SEs.

For the subjective ratings of the target visibility, different stimulus features might have been used by participants, for example, the perceived luminance, flicker, or shape. However, the visibility of all of these features is not necessarily equally influenced by both masking and our singleton manipulation (cf. Breitmeyer et al., 1984). Naive participants do not necessarily have conscious access to the exact perceptual strategy they use for evaluating target visibility. Hence, methods to extract such meanings are based on a number of additional and only approximately met assumptions (cf. Koster et al., 2020). Therefore, we chose to discriminate strategies *post hoc* by looking at the shapes of distributions of individual ratings. Experiments 1 and 2 showed us that the visibility of the targets’ gap location is influenced by our singleton manipulation and, additionally, leads to a characteristic u-shaped function of visibility depending on the different SOA steps (only significant in Experiment 1). Therefore, we used this u-shaped function in the subjective ratings as a benchmark to identify those participants who probably (also) used the gap location of the targets as a basis for their ratings: We fitted a u-shaped function (quadratic) to the visibility depending on the SOA steps separately per person. Only participants with an *R*^2^ > 80% were included in the analyses (15 participants; Figure 8). Note that this selection of participants was conducted based on the u-shape of the functions only: This selection was, thus, blind to the singleton-configuration effect.

**FIGURE 9:**
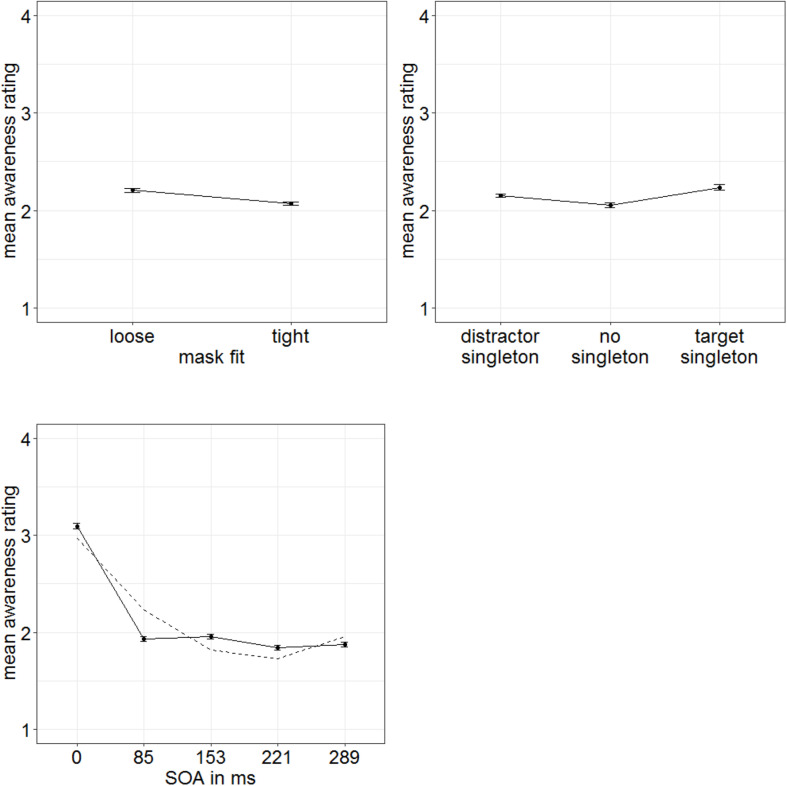
Mean awareness ratings of Experiment 3 (low-awareness group; eight participants) depending on the variables mask fit **(top left panel)**, singleton configuration **(top right panel)**, and stimulus-onset asynchrony (SOA; **bottom left panel**). Error bars represent average SEs.

In order to examine influences of stimulus-driven attention capture especially for cases with lowest target awareness to rule out artifacts caused by residual awareness (as we did in Experiment 1), we extracted a low-awareness group (ratings below median; eight participants). For this group, we found significant main effects for all three variables in the ANOVA (degrees of freedom Greenhouse–Geisser corrected, when necessary): SOA, *F*(1.64,11.46) = 42.83, *p* < 0.001, $\eta_{\text{p}}^{2}$ = 0.86; mask fit (tight: 2.07; loose: 2.20), *F*(1,7) = 16.69, *p* = 0.005, $\eta_{\text{p}}^{2}$ = 0.70; and singleton configuration, *F*(2,14) = 7.29, *p* = 0.007, $\eta_{\text{p}}^{2}$ = 0.51 (Figure 9). No further effects were significant, all *F*’s < 1.51, all *p*’s > 0.239, all $\eta_{\text{p}}^{2}$s < 0.18. To examine the influence of singleton configuration, we compared respective ratings in *post hoc t*-tests (Bonferroni-corrected for three comparisons; α = 0.017). Participants rated their subjective awareness of the targets as singletons (mean rating: 2.23) significantly better than their subjective awareness of the targets presented together with a distractor singleton (mean rating: 2.15), *t*(7) = 3.24, *p* = 0.014, *d* = 1.15, or with no singleton present (mean rating: 2.05), *t*(7) = 3.22, *p* = 0.015, *d* = 1.14. The difference between no singletons and distractor singletons was not significant, *t*(7) = 1.77, *p* = 0.119, *d* = 0.63 (Figure 9, right panel). For a complete ANOVA, with awareness level as between-participants variable (and the within-participants variables SOA, mask fit, and singleton configuration), see Appendix C.

### Discussion

In Experiment 3, we wanted to take a closer look at influences of stimulus-driven attention on subjectively perceived awareness. In line with our assumption that objective discrimination can be used as a proxy for subjectively rated awareness, the manipulation of stimulus-driven capture (by color singletons) influenced awareness judgments in Experiment 3 in the same way that it influenced visibility/objective discrimination performance in Experiments 1 and 2. In addition, we confirmed an independence of the attentional effects from the exact level of awareness by showing a significant singleton-configuration effect even in the below-average awareness group.

In contrast to Experiments 1 and 2, we did find a significant difference in target visibility depending on the distance between stimuli and masks (better visibility with loosely than tightly fitting masks; cf. Bridgeman and Leff, 1979; Schmidt et al., 2006). This was very likely due to some perceptual criterion that was used by our participants for their subjective awareness ratings that was *not* based on the perception of the target’s gap location. For example, participants might have seen less flicker or less target luminance under conditions with closely surrounding masks than under conditions with loosely surrounding masks (cf. Bridgeman and Leff, 1979), and such a perceptual feature could have filtered into the participants’ subjective awareness ratings (despite our usage of a similarity of the awareness ratings to the u-shaped masking function that we observed with target-gap discriminations in Experiment 1).

## General Discussion

Based on contradictory results regarding the relation between attention and awareness, we took this relation to another test using a different approach: We combined stimulus-driven attentional capture by color singletons and a manipulation of visibility and awareness by metacontrast masking. In Experiment 1, we replicated the characteristic u-shaped distribution of ACCs reflecting different levels of stimulus visibility (or perceptual awareness, see Experiment 3), depending on the SOA variation. In addition, as salient stimuli can capture attention in a stimulus-driven way (Theeuwes, 1992; Weichselbaum and Ansorge, 2018), we expected better target visibility (or higher awareness of the targets) in target-singleton than in distractor-singleton conditions. This was exactly what we found. In two experiments, judgments regarding the location of a missing minor sector (the “gap”) of the target disk were better when the target was a singleton than when a distractor away from the target was a singleton. In Experiment 1, a condition without singletons but with local color contrasts at all stimulus positions led to a performance in between the target-singleton and the distractor-singleton condition, demonstrating that it is not the local color difference *per se* that affected stimulus awareness but rather the salience of this local color contrast relative to the lacking color contrasts between adjacent stimuli at other locations (cf. Itti and Koch, 2001). With our salience manipulation, we took great care to rule out alternative, non-attentional explanations. For our salience manipulation, we varied the local contrast in chroma between adjacent stimuli but kept the local luminance contrast the same. In this way, we intended to rule out that principles, such as lateral inhibition in a boundary contour system (Francis, 1997), could explain the interaction of salience and perceptual awareness as well as attention did.

In addition, to measure the capture of attention independently of the masking strength, in Experiment 2, we used the indicators pointing to the target position as probes. We reasoned that capture by singletons should affect not only target visibility or awareness but also the responses to the probes. As target indicators were closer to the target than to the distractors, capture by the singletons should facilitate perception of indicators (as probes) in target-singleton conditions compared to distractor-singleton conditions. This was exactly what we found.

In contrast to our expectations, however, the u-shaped function of target visibility as a function of target-mask SOA (Alpern, 1953; Francis, 1997) was found only in Experiment 1 but not in Experiment 2. We think that the fact that two tasks had to be performed in each trial of Experiment 2—a response to the indicator as a probe and a following judgment about the target—decreased target discrimination performance for the primary-task targets across SOAs, with the effect of flattening the u-shaped metacontrast function. In line with this assumption, accuracy in most intermediate SOAs of Experiment 2 was close to chance (i.e., close to 50%).

In both experiments, in line with an influence of stimulus-driven attention capture that was independent of target awareness and could have even preceded target awareness, we found no interaction between the variables SOA and singleton configuration. However, as it is unclear if the singleton-configuration effect depended on an on average residual target-discrimination ability and, hence, on some visibility/awareness of the target orientation, in Experiment 1, we also took a closer look at the singleton-configuration effect under the conditions of least target visibility (i.e., at an SOA = 85 ms). In line with an awareness-preceding and thus awareness-modulating effect of the singleton configuration, we found that target singletons facilitated correct RTs compared to distractor singleton RTs even at the point of zero discrimination of the target orientations, when we linearly regressed the individual singleton-configuration RT effects on the individual target-orientation discrimination performance in the neutral SOA 85-ms condition. In addition, this linear regression has no significant slope, indicating that additional visibility of the targets did not increase the singleton-configuration effect further.

Note that this does not mean that the color-singleton configuration (of the target vs. the distractor) itself was invisible or that stimulus-driven attention capture was triggered by a subliminal feature itself. This was not tested, as a corresponding test would have required asking our participants to localize the color singletons. Although past studies have sometimes demonstrated strong metacontrast masking of colors, too (Schmidt, 2002), given that we found stimulus-driven attention capture effects but that stimulus-driven capture of attention by subliminal stimuli itself is sometimes not found (cf. Ansorge et al., 2010) and sometimes even leads to a reversed effect (compared to stimulus-driven capture by a supraliminal stimulus; Herreros et al., 2017), we think that it is more likely that in the present study participants have seen the color singleton configuration itself. All that the present results show is that stimulus-driven capture was suited to modulate the participants’ perception and their awareness of the gap orientation of the targets.

The latter was also confirmed in Experiment 3, where we investigated and showed that the singleton configuration not only influenced objective target discrimination in the predicted way, but where it was also demonstrated that target singletons increased the participants’ awareness of the masked targets. Again, this influence was independent of and additive to the influence of SOA, this time on subjective ratings of target awareness. This result demonstrated that the singleton-configuration effect in the objective target-discrimination task that we found in Experiments 1 and 2 did not simply reflect more or less correct guesses under otherwise similarly unaware conditions. Again, however, one should bear in mind that the found independent effects of singleton configurations and of masking on visibility and awareness reflected processing of different features—colors for stimulus-driven attention capture and shapes for masking. Where a similar independence of the effects of stimulus-driven attention and masking on awareness could be found where more related features from the same dimension would be used cannot be concluded from the present data.

Of minor importance, at variance with our expectations, we were only able to demonstrate an influence of spatial target-mask separation, another hallmark of metacontrast masking (Bridgeman and Leff, 1979; Francis, 1997), in Experiment 3 with a subjective measure of awareness. Although our target-mask separation manipulation was as in prior experiments, in which separation had a significant effect on metacontrast masking (Bridgeman and Leff, 1979), our task in Experiments 1 and 2 might have been insensitive to the manipulation of target-mask separation. In fact, assuming that in Experiment 3 the participants used visual criteria such as luminance or flicker besides target-gap location visibility for their subjective awareness ratings, an influence of the task appears as a likely reason for why we were not able to observe the typical influence of target-mask distance in our Experiments 1 and 2. Participants would have simply not used seen flicker or luminance for their reports of the gap locations of the targets.

### Relationship to Prior Research on Stimulus-Driven Attention Capture by Masked Stimuli

Although we pointed out that a variety of studies demonstrated that even stimuli the participants were unaware of (subliminal stimuli) can capture attention in a stimulus-driven way (e.g., McCormick, 1997; Mulckhuyse et al., 2007), this was typically not found with metacontrast-masked stimuli. When metacontrast masking was used to present visual stimuli (e.g., cues) below the threshold of awareness, stimulus-driven salience was insufficient to capture attention (Ansorge et al., 2010; Held et al., 2010; see also Scharlau and Ansorge, 2003). For example, having participants search for one target color (e.g., red), color salience based on a color different from the target color (e.g., a green cue among blue distractors) did not lead to attention capture (Ansorge et al., 2010). However, several differences between these prior studies and the current experiments might account for the results. Most importantly, in the current study, we used liminal rather than subliminal stimuli—that is, our color singletons were visible to some extent at least with the shortest and longest target-mask SOAs. Where there was seemingly no residual awareness of the singletons (i.e., in the medium range of SOAs of Experiment 2), the dual-task situation had probably led us to underestimate the true residual visibility (compared to performance in Experiment 1, where the secondary task was missing). Thus, it is questionable if even the capture of attention by the masked singletons in the intermediate SOA conditions of Experiment 2, which was reflected in the validity effects of the probe discriminations, reflected true instances of completely subliminal salience. As pointed out above, however, this is not entirely certain, as a corresponding safer conclusion would have required to also testing participants’ ability to locate the masked color singletons.

### Debate on Attention Influencing Iconic Memory

Our results let us also draw some conclusions on the relation between iconic memory and stimulus-driven attention. Some researchers support the idea that attention is necessary for creating iconic memory representations (Mack et al., 2015, 2016, 2019), whereas others see them as independent processes (Bachmann and Aru, 2015, 2016; Aru and Bachmann, 2017a,b). As the four relevant stimuli we used in our experiments were within the capacity span found by Sperling (1960), and the timepoint of the target report was earlier than the onset of memory decay (300 ms; cf. Averbach and Coriell, 1961; Jackson-Nielsen et al., 2017), in clearly visible conditions, participants should have been able to remember all four possibly relevant stimuli and report the gap location of the single target indicated by the post-cue. Normally, small parts of the iconic memory that were recognized as being important are transferred to other STM stores such as the visual STM or working memory within the 100 ms before decay. In metacontrast masking, the mental image of the stimulus is replaced by the mental image of the mask presented shortly afterward and therefore hinders the visual input from transferring and transforming into other—more stable and longer lasting—memory representations (Averbach and Coriell, 1961). The u-shaped distribution of ACC depending on the different SOAs between stimuli and masks in Experiment 1 reflects the respective time the information had (or had not) to be transferred to other memory stores before its decay. For SOAs with a higher stimulus visibility (simultaneous presentation and long SOA), the memory representation was already transferred to more durable representations, while in the SOAs with intermediate length, the content of iconic memory had no chance to reach more durable representations before its decay because of the replacing image of the masks. As our singleton variation (stimulus-driven attention) was visible not only in the SOA steps with high stimulus visibility, but also in the short SOA conditions reflecting storage in iconic memory alone, we can conclude that stimulus-driven attention can indeed influence very early memory processes, such as the readout and selection of iconic memory items for working memory. However, Hanning et al. (2015) found an advantage for iconic memory storage due to stimulus-driven attention only, when participants could make eye movements during stimulus presentation. Therefore, one might assume that eye movements are crucial for stimulus-driven attention to influence iconic memory representations. However, the missing effect in Hanning et al. (2015) condition without eye movements might as well be due to their long time-interval between stimulus presentation and report. Although their post-cue appeared already after 300 ms (where we would still expect advantages to show in ACCs), a minimum of 2,100 ms passed before participants could respond. This is problematic, as stimulus-driven capture of attention tends to lose its impact very fast (e.g., Donk and van Zoest, 2008). Additionally, the trace in iconic memory is long gone after 2,100 ms. Thus, with the design by Hanning et al. (2015), it is impossible to tell if stimulus-driven attention had influenced iconic memory prior to its decay.

Another interesting conclusion relates to the ongoing debate about the applicability of iconic memory as a marker for initial awareness-independent representations (Lamme, 2003; Koch and Tsuchiya, 2007). According to our results, showing that potential influences of attention on iconic memory representations are not always reflected in stimulus awareness, iconic memory investigations are not the recommended way to study awareness-related mechanisms.

## Conclusion

We demonstrated the independence of stimulus-driven attention and stimulus visibility/awareness with a new approach combining stimulus-driven capture by color singletons and metacontrast-masking to manipulate visibility/awareness. Although some past evidence for interactions between visual salience and the level of visibility/stimulus awareness in metacontrast masking could have arguably reflected non-attentional influences (e.g., search-asymmetry effect; Tata, 2002), the same cannot be said of the current study, in which alternative explanations in terms of lateral interactions between neuronal activities were ruled out. In addition, we avoided prior complications such as design-related boosts for attentional influences on awareness.

## Data Availability Statement

The raw data supporting the conclusions of this article will be made available by the authors, without undue reservation.

## Ethics Statement

Ethical review and approval was not required for the study on human participants in accordance with the local legislation and institutional requirements. The patients/participants provided their written informed consent to participate in this study.

## Author Contributions

DB, FG, and UA designed the experiments. DB collected and analyzed the data. DB and UA wrote the manuscript. All the authors contributed to the article and approved the submitted version.

## Conflict of Interest

The authors declare that the research was conducted in the absence of any commercial or financial relationships that could be construed as a potential conflict of interest.
