# Supplementary material for: Awareness and Stimulus-Driven Spatial Attention as Independent Processes
Source: Front Hum Neurosci. 2020 Sep 2;14:352. doi: 10.3389/fnhum.2020.00352 (PMC7493193; doi:10.3389/fnhum.2020.00352)

Appendix A

Detailed listing of all significant results of the ANOVAs calculated for Experiments 1 and 2.

Table A1

All Main Effects and Interactions of the Analyses of Variance with Mean Accuracies of the Discrimination of the Missing Minor Sectors of the Target Disks in Experiments 1 and 2

Main effects and interactions	*df*	*F*	*p*	η_p^2
Experiment 1				
SOA	4, 96	10.06	< .001	.30
Mask Fit	1, 24	0.83	.372	.03
Singleton Configuration	1.31, 31.52	14.03	< .001	.37
SOA × Mask Fit	4, 96	1.81	.132	.07
SOA × Singleton Configuration	5.18, 124.37	1.43	.218	.06
Mask Fit × Singleton Configuration	2, 48	0.54	.589	.02
SOA × Mask Fit × Singleton Config.	4.72, 113.36	1.16	.334	.05
Experiment 2				
SOA	4,76	0.59	.669	.03
Mask Fit	1, 19	0.03	.856	< .01
Singleton Configuration	1, 19	7.27	.014	.28
SOA × Mask Fit	2.69, 54.82	0.17	.909	< .01
SOA × Singleton Configuration	4, 76	0.39	.818	.02
Mask Fit × Singleton Configuration	1, 19	0.01	.931	< .01
SOA × Mask Fit × Singleton Config.	4, 76	1.78	.141	.09

Note: Degrees of freedom Greenhouse-Geisser-corrected where necessary.

Table A2

All Main Effects and Interactions of the Analyses of Variance with Mean Reaction Times (RTs) and Mean Accuracies (ACCs) of the Discrimination of the Indicator Line Type in Experiment 2

Main effects and interactions	*df*	*F*	*p*	η_p^2
RT analysis				
SOA	4, 76	5.08	.001	.21
Mask Fit	1, 19	1.20	.287	.06
Singleton Configuration	1, 19	0.75	.396	.04
SOA × Mask Fit	4, 76	0.88	.477	.04
SOA × Singleton Configuration	4, 76	0.11	.978	.01
Mask Fit × Singleton Configuration	1, 19	1.77	.199	.09
SOA × Mask Fit × Singleton Config.	4, 76	1.09	.370	.05
ACC analysis				
SOA	4, 76	2.24	.073	.11
Mask Fit	1, 19	0.00	.945	< .01
Singleton Configuration	1, 19	16.14	< .001	.46
SOA × Mask Fit	4, 76	0.95	.440	.05
SOA × Singleton Configuration	4, 76	0.69	.598	.04
Mask Fit × Singleton Configuration	1, 19	0.42	.524	.02
SOA × Mask Fit × Singleton Config.	4, 76	1.08	.374	.05

Note: Degrees of freedom Greenhouse-Geisser-corrected where necessary.

Appendix B

Analysis of the reaction times (RTs) to the missing minor sectors (or gaps) of the target disks in Experiment 1.

We instructed participants not only to respond accurately, but also quickly. Therefore, we additionally analyzed the RTs to the missing minor sectors of the target disks in Experiment 1 exploratively. Trials with incorrect responses as well as RTs ± 2 *SD*s from the median per person per condition were excluded. To check for influences of the different colors, we compared RTs to green versus red stimuli in a *t* test (red: 611 ms, green: 612 ms). There was no significant difference, $t(24) = 0.16$, $p = .876$, $d = 0.03$. The ANOVA with three within-participant variables, SOA (0 ms/85 ms/153 ms/221 ms/289 ms), Mask Fit (tight/loose), and Singleton Configuration (target singleton/distractor singleton/ no singleton), yielded a main effect for both SOA, $F(4, 96) = 11.56$, $p < .001$, $\eta_p^2 = .33$, and Singleton Configuration, $F(2, 48) = 32.65$, $p < .001$, $\eta_p^2 = .58$ (see Figure B1). The degrees of freedom were corrected using the Greenhouse-Geisser procedure due to a violation of sphericity.

Figure B1

Mean correct reaction times (RTs, in ms) of discriminating the location of the missing minor sectors (or gaps) of the target disks in ms in Experiment 1, depending on the variables Singleton Configuration (left panel) and Stimulus Onset Asynchrony (SOA) steps (right panel). Error bars represent average *SE*s.

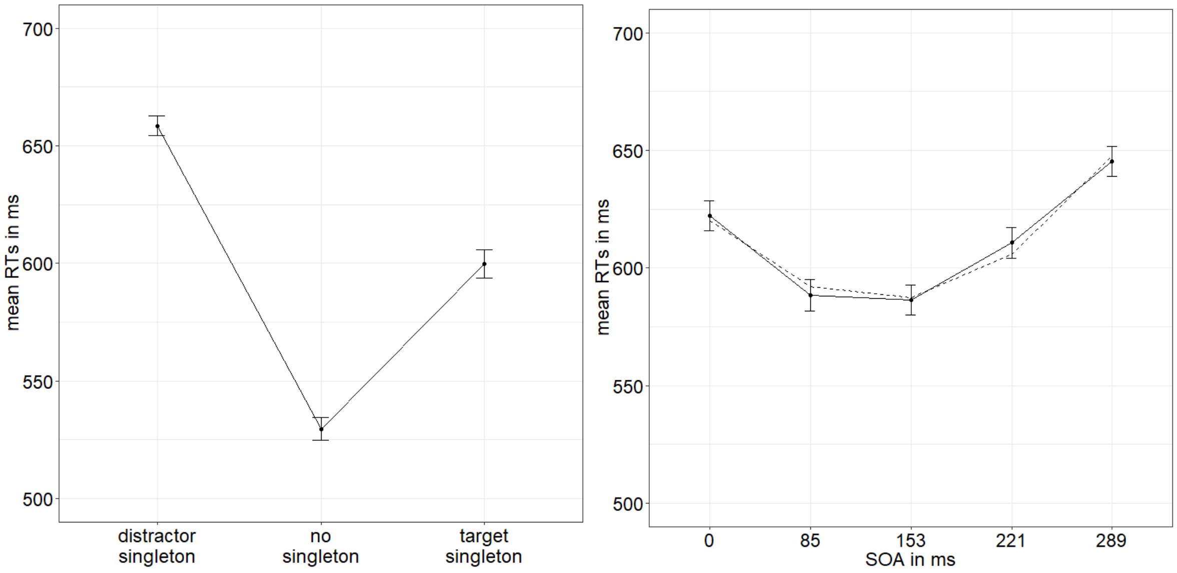

Appendix C

Analysis of the mean awareness ratings in Experiment 3, with three within-participant variables (SOA, Mask Fit, and Singleton Configuration) and the between-participants variable Awareness Level (high vs. low).

We found significant main effects for Awareness Level (reflecting our grouping into low- and high-awareness groups), SOA, Mask Fit, and Singleton Configuration (see Table C1). These effects were all as expected. In addition, the interaction between SOA and Singleton Configuration was significant (see Table C1). Figure C1 shows that this was not particularly interesting (the effect goes back to the longest SOA step, where the difference between target and distractor singletons is no longer present). Finally, a significant interaction between Awareness Level, Mask Fit, and Singleton Configuration (see Table C1) is visualized in Figure C2. Note that the low-awareness group (in contrast to the high-awareness group) shows different ratings for the different singleton configurations, which rules out the possibility of this effect being due to residual target awareness.

Table C1

All Significant Main Effects and Interactions of the Analyses of Variance with Mean Awareness Ratings in Experiment 3

Significant main effects and interactions	*df*	*F*	*p*	η_p^2
Awareness Level	1, 13	12.22	.004	.48
SOA	1.94, 25.21	66.26	< .001	.84
Mask Fit	1, 13	32.96	< .001	.72
Singleton Configuration	1.13, 14.47	6.70	.018	.34
SOA × Singleton Configuration	3.02, 39.23	3.39	.027	.21
Awareness Level × Mask Fit × Singleton Configuration	2, 26	4.05	.029	.24

Note: Degrees of freedom Greenhouse-Geisser-corrected where necessary.

Figure C1

Mean awareness ratings in Experiment 3 depending on the variables Singleton Configuration and Stimulus Onset Asynchrony (SOA). Error bars represent average *SE*s.

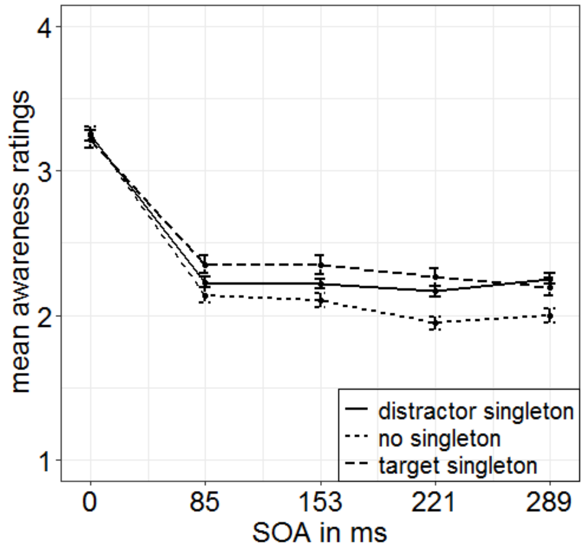

Figure C2

Mean awareness ratings in Experiment 3 depending on the variables Singleton Configuration and Mask Fit, separately for high awareness (left panel) and low awareness (right panel). Error bars represent average *SE*s.

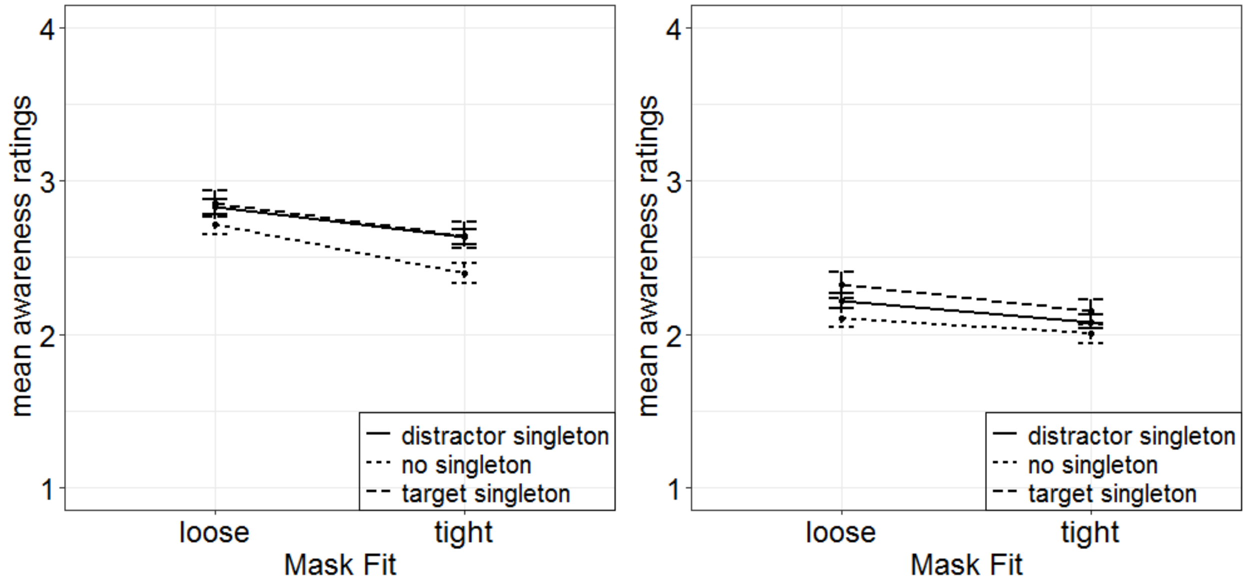

Supplement: Supplementary file 1 [file Data_Sheet_1.pdf]
